# Bacterial contamination of neonatal intensive care units: How safe are the neonates?

**DOI:** 10.1186/s13756-021-00901-2

**Published:** 2021-01-30

**Authors:** Dharm Raj Bhatta, Supram Hosuru Subramanya, Deependra Hamal, Rajani Shrestha, Eva Gauchan, Sahisnuta Basnet, Niranjan Nayak, Shishir Gokhale

**Affiliations:** 1 Department of Microbiology, Manipal College of Medical Sciences, Pokhara, Nepal; 2 Department of Pediatrics and Neonatology, Manipal College of Medical Sciences, Pokhara, Nepal

**Keywords:** NICU, Nosocomial infections, Bacterial contamination, Disinfection, Antibiotic resistance

## Abstract

**Background:**

Intensive care units (ICU) are essential healthcare facility for life threatening conditions. Bacterial contamination of objects/instruments in ICU is an important source of nosocomial infections. This study is aimed to determine the level of bacterial contamination of instruments/objects which are commonly touched by healthcare workers and frequently come in contact with the neonates.

**Methods:**

This hospital based prospective study was conducted in neonatal intensive care unit (NICU) of Manipal Teaching Hospital, Pokhara, Nepal. A total of 146 samples collected from surfaces of incubators, radiant warmers, suction tips, ventilators, stethoscopes, door handles, weighing machines, mothers’ beds, phototherapy beds, laryngoscope, telephone sets, blood pressure machine, etc. formed the material of the study. Isolation, identification and antibiotic susceptibility of the bacterial isolates was performed by standard techniques. Blood culture isolates from NICU patients during the study period were compared with the environmental isolates.

**Results:**

Out of 146 samples, bacterial growth was observed in 109. A total of 119 bacterial isolates were retrieved from 109 samples. Three common potential pathogens isolated were *Escherichia coli* (n = 27), *Klebsiella* species (n = 21) and *Staphylococcus aureus* (n = 18). Majority of *E. coli* and *Klebsiella* isolates were from incubators, suction tips and mothers’ beds. Majority of *S. aureus* isolates were cultured from radiant warmers. Among *S. aureus* isolates, 33.3% (6/18) were methicillin resistant. Majority of the bacterial isolates were susceptible to gentamicin and amikacin. Common potential pathogens isolated from blood culture of NICU patients were *S. aureus* and *Klebsiella* species*.*

**Conclusion:**

High degree of bacterial contamination of objects/instruments in NICU was recorded. Isolation of potential pathogens like *E. coli*, *Klebsiella* species and *S. aureus* is a major threat of nosocomial infections. Blood culture data of NICU reflects possibility of nosocomial infections from contaminated sites. Gentamicin and amikacin may be used for empirical therapy in suspected cases of nosocomial infections in NICU.

## Background

Intensive care units (ICU) are essential components of healthcare facility for treatment of life threatening conditions. Low birth weight infants admitted in neonatal intensive care unit (NICU) are immunocompromised and vulnerable to nosocomial infections [1, 2]. Bacterial contamination of objects/instruments in ICU is a major source of nosocomial infections. Every year, more than one million neonatal deaths are reported worldwide [3]. Nosocomial infections among neonates are responsible for 30–40% of the death in resource constrained countries [4, 5]. Bacteria, fungi and viruses contaminate and survive on the surfaces, equipment and indoor environment of NICU for variable duration [6]. Colonized healthcare workers (HCW) and patients are other important sources of pathogens. The contributory factors include poor hand hygiene, overcrowding, understaffing, inadequately trained staff and insufficient disinfection/fumigation. Approximately one-third of nosocomial infections can be prevented by adapting strict infection control protocols [7].

Neonates admitted in NICU are vulnerable to nosocomial infections emanating from contaminated objects/instruments. The risk of transmission is higher during surgical and mechanical manipulations such as arterial and venous catheters, tracheal cannulas, peritoneal shunt, chest drains etc. [8, 9]. Various bacterial agents have been implicated in contamination of ICU. Clinically important potential pathogens include *S. aureus* including methicillin resistant *S. aureus* (MRSA), *Klebsiella* species, *E. coli*, *Pseudomonas* species, *Acinetobacter* species and *Enterococcus* species. [10]. Increasing trends of antimicrobial resistance and emergence of multidrug resistant (MDR) pathogens like MRSA, vancomycin resistant *S. aureus* (VRSA), extended spectrum beta lactamase (ESBL) producing *Enterobacteriaceae* and *Acinetobacter* species in NICU result into high morbidity and mortality.

The spectrum of organisms colonizing NICU environment changes over time and varies from hospital to hospital within and outside country. Therefore, this study was undertaken to evaluate the bacterial contamination of commonly touched objects/instruments of NICU. Despite increasing incidences of nosocomial infections, few studies have explored the role of bacteriological contamination of NICU surfaces. Contamination monitoring in NICU is rare in hospitals of Nepal and limited data is available. This probably is the first study from Nepal to report bacteriological profile and antibiotic resistance patterns of environmental isolates of NICU. Present study was aimed to determine the level of bacterial contamination of the instruments/objects commonly touched by HCW and/or which frequently come in contact with neonates. Bacteriological examination of NICU environmental samples could provide information about level of bacterial contamination, antibiotic resistance patterns of the isolates and effectiveness of cleaning/disinfection procedures.

## Methods

This hospital based prospective study was conducted in NICU of Manipal Teaching Hospital, Pokhara over a period of ten months. Approval from the Institutional Review Committee (IRC) of Manipal College of Medical Sciences, Pokhara, Nepal, was obtained before commencement of the study (MEMG/IRC/311/GA). Manipal Teaching Hospital is a 750 bedded tertiary care hospital of Western Nepal, with Medical ICU, Neuro ICU, Surgical ICU, Critical Care Unit, Pediatric ICU and NICU. This hospital has well equipped NICU to accommodate 21 neonates. The detailed information regarding cleaning/disinfection of objects/instruments of NICU were obtained. Healthcare professionals of NICU follow standard hand washing protocols before and after examination of each baby. The floor is mopped twice a day with detergent solution. Non invasive objects/instruments, table tops and other surfaces are wiped with 70% isopropyl alcohol swabs. Invasive instruments are sterilized/disinfected by autoclaving and/or Cidex as per manufacturer’s directions. NICU is fumigated once in three months by fumigator filled with quaternary ammonium compound solution (SOT 125 TM).

### Specimen collection

A total of 146 samples were collected from surfaces of incubators, radiant warmers, suction tips, ventilators, stethoscopes, ambu bags, door handles, digital weighing machines, mothers’ beds, phototherapy beds, laryngoscope, bedside locker, telephone sets, hood box, blood pressure machine, station counter, wall BPL monitor and sterilizer. Majority of these sites either come in direct contact with healthcare professionals or neonates. Samples were collected by rubbing sterile swabs moistened with peptone water.

### Isolation and identification of bacterial isolates

Samples were inoculated immediately in peptone water and incubated overnight. Sub cultures were performed on MacConkey agar and Blood agar plates. Plates were incubated aerobically at 37 °C for 24–48 h. Identification of the isolates was performed by standard microbiological techniques such as colony morphology, microscopic features and standard phenotypic characters [11].

### Antibiotic susceptibility test

Antibiotic susceptibility testing of the isolates was performed on Mueller Hinton agar (HI media, Mumbai, India) by Kirby Bauer disc diffusion method [12]. Bacterial isolates showing resistance to at least one agent in three or more antimicrobial categories were labeled as MDR [13]. Methicillin resistance among *S. aureus* was detected by cefoxitin (30 µg) disc diffusion method [12]. The ESBL production by Gram-negative bacilli was detected by standard methods [12]. Details of isolates from the blood culture of the patients admitted in NICU during the study period were extracted from medical record and compared with environmental isolates of this study.

### Biofilm detection

Biofilm forming ability of the *S. aureus* isolates was tested by Congo red agar test method [14]. Isolates with black colonies were reported as biofilm producers.

## Results

Out of 146 samples collected from various sites, bacterial growth was observed in 109 specimens while 37 samples did not show bacterial growth. A total of 119 bacterial isolates were retrieved from 109 samples. Details of sampling sites and bacterial isolates are depicted in Table 1. Three potential pathogens isolated were *E. coli* (27/119), *Klebsiella* species (21/119) and *S. aureus* (18/119). Majority of *E. coli* and *Klebsiella* isolates were isolated from incubators, suction tips and mothers’ beds. Majority of *S. aureus* isolates were cultured from radiant warmers. Among *S. aureus* isolates, 33.3% (6/18) were MRSA and remaining were MSSA. Vancomycin resistant *S. aureus* (VRSA) was not detected. Other bacterial isolates were *Acinetobacter* species, *Pseudomonas* species, Coagulase negative *Staphylococci*, *Enterococcus* species, *Micrococcus* species, Diphtheroid and aerobic spore bearers. The antibiotic resistance patterns of bacterial isolates are shown in Table 2.

**Table 1:** Bacteria isolated from NICU environmental surfaces

Sampling sites	Bacterial isolates						
	*E. coli*	*Klebsiella* species	*Pseudomonas* species	*Acinetobacter* species	*S. aureus*	CoNS	*Enteroccus* species
Radiant warmer	02	02	–	01	12	–	–
Suction tip	06	04	06	02	–	–	02
Incubator	08	02	01	–	–	04	01
Stethoscope	–	–	–	–	01	02	02
Phototherapy bed	02	01	–	–	–	–	–
Mothers’ bed	05	04	01	02	–	–	–
Ambu bag	–	02	–	–	–	–	–
Ventilator	01	–	–	–	–	–	–
Door handles	–	–	–	–	02	01	03
Weighing machine	01	03	–	–	–	02	01
Laryngoscope	01	–	–	–	02	01	–
Telephone set	–	–	–	01	–	02	02
Bedside locker	01	01	–	–	–	02	02
BP Machine	–	–	–	01	–	03	02
Station counter	–	01	–	–	–	01	–
Hood box	–	–	–	–	–	02	–
Wall BPL monitor	–	01	01	–	–	02	–
Sterilizer	–	–	–	–	01	–	–
Total	27	21	09	07	18	22	15

**Table 2:** Antibiotic resistance pattern of bacterial isolates

Antibiotic	*E. coli* (N = 27) Frequency (%)	*Klebsiella species* (N = 21) Frequency (%)	*Pseudomonas* species (N = 09) Frequency (%)	*Acinetobacter* species (N = 07) Frequency (%)	*S. aureus* (N = 18) Frequency (%)
Ampicillin	27 (100)	21 (100)	–	–	–
Ciprofloxacin	8 (29.6)	7 (33.3)	4 (44.4)	3 (42.8)	6 (33.3)
Gentamicin	10 (37)	11 (52.4)	2 (22.2)	2 (28.5)	1 (5.5)
Ceftazidime	22 (81.5)	18 (85.7)	4 (44.4)	3 (42.8)	–
Ceftriaxone	21 (77.7)	17 (80.9)	–	–	–
Co-trimoxazole	9 (33.3)	6 (28.5)	–	–	5 (27.7)
Amikacin	0	0	0	0	0
Imipenem	0	0	0	0	–
Cefoxitin	–	–	–	–	6 (33.3)
Erythromycin	–	–	–	–	11 (61.1)
Clindamycin	–	–	–	–	04 (22.2)

Majority of the bacterial isolates were susceptible to imipenem, gentamicin and amikacin. High percentage of multidrug resistance was observed among *E. coli* 37% (10/27) and *Klebsiella* species 52.4% (11/21). Out of 18 *S. aureus* isolates, 27.7% (5/18) were MDR and 33.3% (6/18) were biofilm producers. Biofilm production among MRSA (66.6%) isolates was significantly higher than MSSA (16.6%) isolates (*p* value < 0.001). Among Gram-negative bacteria, majority of *E. coli* 70.3% (19/27) and *Klebsiella* species 71.4% (15/21) were ESBL producers which is significantly higher than non fermentative Gram-negative bacilli (*p* value < 0.01). Details of ESBL production among Gram-negative isolates is depicted in Table 3.

**Table 3:** ESBL production status of the Gram-negative isolates

Organisms	ESBL		Total
	ESBL producers (%)	ESBL non producers (%)	
*E. coli*	19 (70.3%)	08 (29.7%)	27
*Klebsiella* species	15 (71.4%)	06 (28.6%)	21
*Pseudomonas* species	03 (33.3%)	06 (66.7%)	09
*Acinetobacter* species	03 (42.8%)	04 (57.2%)	07
Total	40 (62.5%)	24 (37.5%)	64

Common isolates from blood culture were *S. aureus* (n = 13) and *Klebsiella* species (n = 5). Eight isolates of *S. aureus* and three isolates of *Klebsiella* species from blood culture have antibiogram similar to that of the environmental isolates.

## Discussion

Nosocomial infections in NICU are one of the most important causes of morbidity and mortality [8, 9]. The mortality rates in NICU of resource constrained countries vary from 11.9 to 14.7%, much higher than the rates in high resource countries (6.1–7.1%) [15–17]. Microbial agents constantly inhabit the hospital environment including NICU. Bacterial contamination of NICU is one of the major factors responsible for higher incidences of nosocomial infections.

High bacterial contamination of frequently touched objects/instruments in NICU was recorded. Overall bacterial contamination rate in NICU was 74.6% (109/146) which is higher than other studies [18, 19]. Similar studies have reported contamination rates ranging from 59.2 to 67.8% [10, 19]. Higher bacterial contamination in NICU may be attributed to admission of neonates with variety of clinical conditions, overcrowded units, faecal contamination, easy access to visitors, understaffing and poor compliance to infection control practices. Prolonged NICU stay necessitates frequent visits by mothers and HCW result into increased human activities facilitating exchange of bacterial flora. Bacterial culture yielded wide variety of organisms ranging from opportunistic to potential pathogens. Common potential pathogens isolated were *E. coli*, *Klebsiella* species and *S. aureus*.

Different members of family *Enterobacteriaceae* colonize NICU and cause neonatal infections [20]. The pathogens most often implicated in neonatal infections in resource constrained countries are *Klebsiella* species,* E. coli*,* Pseudomonas* and *S. aureus* [21]. In our study findings, potential pathogens isolated from various surfaces were *E. coli*, *Klebsiella* species and *S. aureus*. Majority of the *E. coli* and *Klebsiella* species were recovered from incubators and suction tips. *E. coli* is frequently associated with neonatal sepsis and one of the most common causes of acute pyogenic meningitis among neonates. Surface contamination of NICU by *E. coli* and *Klebsiella* species lead to greater risk of systemic infections like neonatal septicemia, pneumonia and meningitis especially among the premature neonates. Contamination of NICU surfaces by Gram-negative bacilli is possibly associated with the faeces of the neonates. Among *E. coli* and *Klebsiella* isolates, 70.3% (19/27) and 71.4% (15/21) were ESBL producers respectively. High percentage of ESBL producing organisms limit the treatment choice and may result into treatment failure.

Another notorious nosocomial pathogen isolated was *S. aureus*. Surfaces of radiant warmers yielded highest number of *S. aureus* and MRSA. Hands of HCW and visitors are the common source of *S. aureus* and MRSA in hospital. Previous studies have documented that hands of HCW account for 20 to 40% infections due to cross-transmission within the units [22, 23]. Isolation of *S. aureus* from the surfaces of radiant warmers, stethoscopes and door handles indicate human hands as important source of *S. aureus* in NICU. Presence of *S. aureus* and MRSA on these surfaces increases the risk of transmission and may subsequently result into sepsis and pneumonia. Among *S. aureus* isolates, 33.3% were MRSA. Neonatal infections with MRSA are difficult to treat, leading to prolonged hospital stay and long term therapy. *S. aureus* has been identified as an important nosocomial pathogen due to its ability to survive on inanimate objects for several days [6]. Biofilm formation helps long term survival of *S. aureus*. Majority of MRSA isolates 66.6% (4/6) in our study were biofilm producers. Previous study from Manipal hospital had reported slightly lower percentage (62.5%) of biofilm among MRSA isolates from environmental surfaces [24]. This finding is alarming as MRSA isolates embedded in biofilm can survive longer and are potential source of nosocomial infections. There are limited data available in this field which studied biofilm production among environmental isolates.

Microbial flora of NICU surfaces is not significantly different from other units of the hospital environments. However, the additional challenge in preventing nosocomial infections in NICU is the high susceptibility of premature and immunocompromised neonates. Colonization of NICU surfaces by opportunistic nosocomial pathogens like *Acinetobacter* species, Coagulase negative *Staphylococci*, *Pseudomonas* species and *Enterococcus* species are important for the high risk neonates such as low birth weight, premature and congenital abnormalities. In this study, we observed high resistance of the bacterial isolates to commonly used antibiotics such as ampicillin, ceftazidime, ceftriaxone and ciprofloxacin. Similar findings have been reported in other studies [10, 25]. High percentage of MDR was observed among both Gram-negative and Gram-positive bacterial isolates. Among Gram-negative bacilli, more than 50% of the isolates were MDR which is alarming. Similarly, 27.7% (5/18) of *S. aureus* isolates were MDR. High percentage of MDR among bacterial pathogens could be attributed to use of higher generation of antibiotics for empirical treatment and use of prophylactic antibiotics for high risk mothers and neonates. Data of antibiotic resistance patterns of bacterial pathogens would help clinicians to formulate empirical antimicrobial therapy in suspected cases of nosocomial infections in NICU. This may help in reducing duration of NICU stay and neonatal mortality. The long term effect will be promoting antimicrobial stewardship.

Blood culture is one the most common microbiological investigations ordered from NICU. Blood culture data of NICU patients revealed that, *S. aureus* and *Klebsiella* species were two most common causes of neonatal sepsis. Similarities in antibiograms were observed among majority of *S. aureus* and *Klebsiella* species isolated from blood culture and in this study of NICU environment. This possibly indicates the nosocomial transmission of these pathogens resulting into sepsis. Comparing antibiotic resistance patterns of environmental isolates and blood culture isolates of NICU is one of the easy phenotypic method. This has been achieved in this study but not by other workers.

Many of the hospitals in resource constrained countries like Nepal, either have no NICU or have single NICU. Because of high bed occupancy in NICU, standard guidelines for cleaning/disinfection are poorly implemented. This results into increased bacterial colonization and subsequent spread within NICU. It is practically difficult to maintain sterility in the NICU environment because of high rate of HCW activities and use of equipments. Meticulous cleaning/disinfection protocols are necessary to prevent the retention and spread of virulent microbial pathogens in sensitive environment of NICU. In this study we have included most of the objects/instruments commonly touched by HCWs and objects which frequently come in contact of neonates. This is an attempt to determine relevance of flora on these objects and their role in nosocomial infections. The findings of this study have provided baseline information about the degree of contamination and resistance patterns of environmental isolates.

Findings of this study are important to generate awareness among healthcare professionals of resource constrained countries regarding contamination of NICU and their possible role in neonatal nosocomial infections. This study will stimulate scientists/researchers of resource constrained countries to explore more about neonatal nosocomial infections and devise possible preventive measures.

### Limitations of the study

Association of potential pathogens isolated from objects/instruments of NICU in sepsis was demonstrated only based on antibiogram of the isolates. Genotypic characterization of the isolates was not performed. Biofilm property was studied only for *S. aureus* isolates. This was single center, time limited study conducted in one tertiary care hospital and results may not be generalized.

## Conclusion

Bacterial contamination of objects/instruments in NICU was high. Isolation of potential pathogens like *E. coli*, *Klebsiella* and *S. aureus* is threat to the neonates. Blood culture data of NICU reflects possibility of nosocomial transmission. This study emphasizes need of suitable decontamination protocols and hand hygiene. Regular surveillance and effective disinfection techniques would reduce the bacterial colonization and transmission to the neonates. Gentamicin and amikacin may be used for empirical therapy in clinically suspected cases of sepsis. Similar studies at different centers are needed for better understanding of nosocomial infections in NICU.

## Data Availability

Contact author for data request.

## Abbreviations

ESBL: Extended spectrum beta lactamases
HCW: Healthcare workers
ICU: Intensive care units
MRSA: Methicillin resistant *Staphylococcus aureus*
MSSA: Methicillin sensitive *Staphylococcus aureus*
MDR: Multidrug resistant
NICU: Neonatal intensive care units
VRSA: Vancomycin resistant *Staphylococcus aureus*
